# In vitro and in vivo anti-piroplasm activity of PBT2 against *Theileria annulata* and *Babesia microti*

**DOI:** 10.1016/j.ijpddr.2026.100646

**Published:** 2026-04-22

**Authors:** Jin Che, Yixuan Wu, Junwei Wang, Yijun Chai, Jinming Wang, Wei Li, Shuaiyang Zhao, Guiquan Guan, Hong Yin

**Affiliations:** a State Key Laboratory of Animal Disease Control and Prevention, Key Laboratory of Veterinary Parasitology of Gansu Province, Lanzhou Veterinary Research Institute, Chinese Academy of Agricultural Sciences, Xujiaping 1, Lanzhou, Gansu, 730046, China; b College of Veterinary Medicine, Northeast Agricultural University, Harbin, 150006, China; c Jiangsu Co-Innovation Center for the Prevention and Control of Important Animal Infectious Diseases and Zoonoses, Yangzhou University, Yangzhou, 225009, China

**Keywords:** *Theileria annulata*, *Babesia microti*, PBT2, Drug resistance

## Abstract

Theileriosis and babesiosis remain major constraints to livestock production, and increasing drug resistance necessitates alternative therapeutic strategies. Here, we evaluated the metal ionophore PBT2 for anti-piroplasm activity. PBT2 inhibited proliferation of two *Theileria annulata* schizont infected cell lines with IC_50_ values of 450.9 nM and 407.8 nM and significantly reduced parasite burden. Colony formation was suppressed in both cell lines, consistent with parasite-associated growth inhibition. Cytotoxicity in BoMAC cells was limited, yielding a selectivity index exceeding 60-fold. ICP-MS analysis of purified schizonts showed dose-dependent Zn accumulation and concomitant Mn depletion following PBT2 treatment, whereas Cu and Fe levels were not significantly altered. In infected cells, PBT2 increased reactive oxygen species levels and reduced total superoxide dismutase activity. Partial restoration of viability by Mn supplementation supports a functional link between Mn depletion and growth inhibition. In a *Babesia microti* murine model, PBT2 reduced peak parasitemia in a dose-dependent manner and ameliorated infection-associated anemia. Our findings indicate PBT2 as a promising, safe, and broad-spectrum anti-piroplasm chemical compound that could be used to develop the drug formulation for controlling the infections of drug resistant piroplasm in future.

## Highlights

• PBT2 demonstrates potent in vitro activity against *Theileria annulata* schizonts, including a buparvaquone-resistant strain.
• PBT2 shows low cytotoxicity in BoMAC cells with >60-fold selectivity, indicating a favorable safety margin.
• PBT2 disrupts metal homeostasis, inducing Zn accumulation, Mn depletion, ROS increase, and reduced SOD activity.
• PBT2 reduces *Babesia microti* parasitemia and alleviates anemia in mice, supporting broad-spectrum efficacy.

## Graphical abstract

**Figure:**
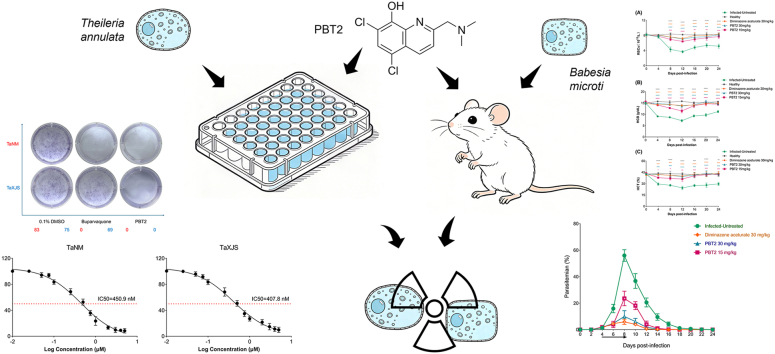

## 1 Introduction

Piroplasms, a group of tick-borne apicomplexan parasites characterized by pear-shaped intraerythrocytic stages, primarily encompass pathogenic *Theileria* spp and *Babesia* spp. Piroplasms belong to the most common group of mammalian blood parasites, infect a wide range of hosts, including humans, cattle, sheep, horses and rodents, causing substantial economic losses globally (Jalovecka et al., 2018; Almazan et al., 2022). The alarming rise of drug resistance due to antiparasitic medicines misuse poses significant challenges to disease control, public health, and sustainable livestock production (Marcos and Wormser, 2023; Rashid et al., 2024). Drug repurposing presents a promising strategy to circumvent this challenge, offering a time and resource efficient pathway to identify novel therapeutic options against resistant parasites infection (Hostettler et al., 2016; Villares et al., 2022; Singh et al., 2025).

PBT2, a derivative of the 8-hydroxyquinoline (8-HQ) scaffold, functions as a Cu/Zn ionophore that facilitates intracellular metal accumulation (Van Zuylen et al., 2021). Previously investigation of PBT2 as a potential therapeutic for neurodegenerative disorders such as Huntington's and Alzheimer's disease had progressed to Phase II clinical trials (Lannfelt et al., 2008; Faux et al., 2010). While its primary neurological efficacy endpoints were not fulfilled, extensive clinical data have established a favorable safety profile for PBT2 both in vitro and in vivo (Lannfelt et al., 2008; Bush, 2013; Adlard et al., 2014).

Zn is indispensable for cellular physiology, serving as a cofactor for a multitude of enzymes and regulatory proteins while it's also acting as a key signaling molecule (Yamasaki et al., 2007; Maret, 2009). However, the biological utility of Zn is a double-edged sword, necessitating imperative and precise control over the intracellular concentration of free Zn^2+^ to prevent the detrimental effects associated with both its depletion and overload (Kim et al., 2000).

In this study, we demonstrate the efficacy of PBT2 against piroplasm parasites. PBT2 effectively inhibits *Theileria annulata* (*T. annulata*) proliferation in vitro and suppresses *Babesia microti* (*B. microti*) infection in vivo, revealing its potential as a novel broad-spectrum anti-piroplasm chemical compound.

## 2 Materials and methods

### 2.1 Parasites and cell culture

TaNM and TaXJS are two lines of *T. annulata* schizont-infected bovine lymphocytes, representing buparvaquone (Bup) susceptible and resistant phenotypes, respectively (Che et al., 2025). TaXJS remained proliferative at Bup concentrations up to 6 μM, whereas TaNM was fully inhibited at 600 nM. BoMac was a bovine macrophage line immortalized by the simian 40 (SV40) large T antigen (Stabel and Stabel, 1995). The above three cell lines were cultured in RPMI 1640 medium, supplemented with 10% fetal bovine serum (FBS) at 37 °C with 5% CO2.

In vivo experiments were conducted by infecting 10-week-old male BALB/c mice with *B. microti* preserved in Vector and Vector-borne Diseases (VVBD) laboratory, Lanzhou Veterinary Research Institute (LVRI), Chinese Academy of Agricultural Sciences (CAAS), China.

### 2.2 Chemicals

**Fig. 1:**
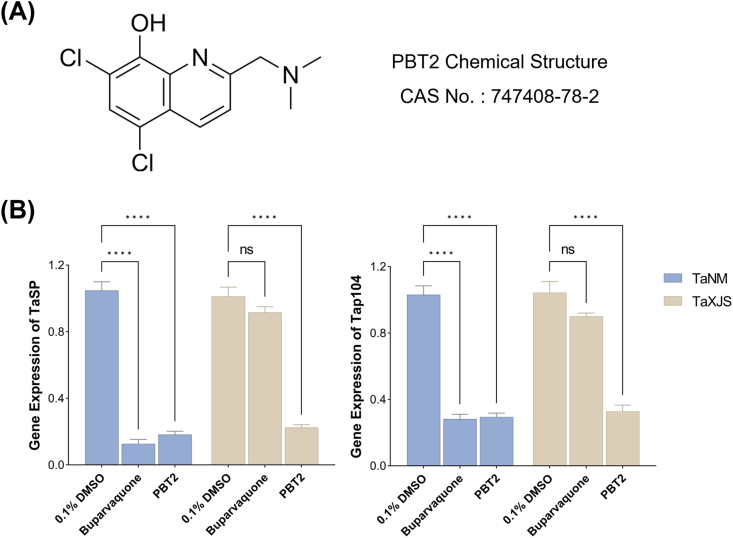
Structures of PBT2 (A) and PBT2 reduce the mRNA levels of *TaSP* and *Tap104* (B). Error bars represent the SD of the mean from three independent biological replicates. ns, *P*> 0.05; ∗∗∗∗, *P* < 0.0001 by two-way ANOVA followed by Dunnett's multiple comparisons test.

The compounds PBT2 (Fig. 1A), Bup and Diminazene aceturate (DA) were purchased from MedChemExpress (MCE) and stored as a 10 mM stock solution in DMSO (Macklin). HNO_3_ was purchased from Merck. The multi-element standard solution containing Cu, Zn, Mn, and Fe (10 mg/mL) for ICP-MS was purchased from Agilent Technologies. MnSO_4_·H_2_O solution purchased from Sigma.

### 2.3 In vitro efficacy of PBT2 on growth of *T. annulata*

TaNM and TaXJS cells were plated in 96-well plate (Bottom Poly-D-Lysine Coated, Corning) with 1.5 × 10^4^ cells per well in 100 μl RPMI 1640 and incubated at 37 °C with 5% CO_2_. The cells were divided into groups and simultaneously treatment for 48 h under respective conditions: PBT2 (1 μM), Bup (600 nM) as a positive control (PC), and 0.1% DMSO as a negative control (NC). Immunofluorescence was performed on fixed cells using a specific anti-H3K18me1 (Abacm 177253) to label the parasite nuclei (Cheeseman et al., 2021) and host nuclei was stained with DAPI. Images were captured and analyzed using EVOS M5000 Imaging System (ThermoFisher Scientific).

### 2.4 Cell viability and proliferation assay in vitro

TaNM, TaXJS and BoMac cells were plated in 96-well plate with 1.5 × 10^4^ cells per well in 100 μl RPMI 1640 and incubated at 37 °C with 5% CO_2_. Different concentrations (0.01-5 μM) of PBT2 were added to wells to incubate for 48 h 10 μl of Cell Counting Kit-8 (CCK-8) (Abbkine, China) were added to each well and incubated for another 4 h. The absorbance at 450 nm was measured using a microplate reader and calculated the cell survival rate.

Clonogenic potential was evaluated using a standard colony formation assay (Franken et al., 2006). Cells (1 × 10^3^ per well) were seeded in 6-well plates and cultured in complete RPMI-1640 medium supplemented with 10% FBS at 37 °C with 5% CO_2_. After 7 - 9 days, colonies were fixed with 4% paraformaldehyde, stained with 0.5% crystal violet, and counted manually. Bup (600 nM) and 0.1% DMSO were used for positive control (PC) and negative control (NC), respectively.

For manganese rescue assays, TaNM cells (1.5 × 10^4^ per well) were seeded in 96-well plates and treated with PBT2 (0.5 μM), Mn (100 μM), or a combination of PBT2 (0.5 μM) and Mn (100 μM) for 48 h. Cell viability was subsequently assessed using a CCK-8 assay.

### 2.5 Immunofluorescence analysis

At the indicated treatment time points, cells were washed 3 times with PBS, with supernatant gently aspirated using a pipette, and then fixed with 4 % paraformaldehyde for 30 min at room temperature. After fixation, the cells were washed 2 times with PBS and permeabilized with 0.5 % Triton X-100 for 5 min. Cells were then washed 3 times with PBS and blocked with 3% bovine serum albumin (BSA) for 1 h at room temperature. Subsequently, cells were incubated with anti-H3K18me1 antibody (0.5 μg/mL) overnight at 4 °C. After 5 times washed with PBS, cells were incubated with Alexa Fluor 488 Goat anti-Rabbit antibody (ThermoFisher Scientific, 0.2 μg/mL) for 1 h at room temperature in dark. Nuclei were counterstained with DAPI (ThermoFisher Scientific, 0.5 μg/mL) for 15 min at room temperature in the dark. Following final PBS washed to remove unbound dye, samples were maintained in PBS and immediately visualized under a fluorescence microscope.

### 2.6 RNA extraction and RT-qPCR

Total RNA was extracted using the RNeasy Mini Kit (Qiagen, Germany) according to the manufacturer's instructions. PrimeScript™ RT Reagent Kit (TaKaRa, Japan) was used for reverse transcribed of 1 μg RNA. The transcription levels of *TaSP* and *TaP104* were analyzed using gene-specific primers (Table 1) (Hostettler et al., 2014). RT-qPCR was performed using TB Green Premix Ex Taq (TaKaRa) according to the manufacturer's instructions with QuantStudio 5 instrument (Applied Biosystems, USA). The amplification program was as follows: initial denaturation at 95 °C for 30 s, followed by 40 cycles of denaturation at 95 °C for 5 s and annealing/extension at 60 °C for 10 s. A melt curve analysis was subsequently performed at 95 °C for 15 s, 60 °C for 60 s, and 95 °C for 15 s.

**Table 1:** Primers used in the present study.

Gene	Primer	Sequence	Reference
Tap104	Tap104-F	TCATAGGTCTACAGAACTGGA	Hostettler et al. (2014)
	Tap104-R	TTTAGGTGGTTCTGGACCCT	
TaSP	TaSP-F	AGCAGCCCCTTGTCATGGG	
	TaSP-R	TAATAGCTTTTGCACGGAGGA	
Actin	Actin-F	GAGACCACCTACAACAGCATCATG	
	Actin-R	CACCTTGATCTTCATGGTGCTGGG	

### 2.7 Purification of the *T. annulata* schizonts

Two concentrations of PBT2 (0.5, 1 μM) were added to TaNM cell to incubate for 24 h. Subsequently, 1 × 10^6^ cells were collected and subjected to *T. annulata* schizonts purification (Arrowood and Sterling, 1987; Regan et al., 1991; Sugimoto et al., 1991). Briefly, infected cells were treated with nocodazole (Sigma, USA) for 16 h to disrupt host microtubule networks, facilitating subsequent parasite release. Cells were then incubated on ice with trypsin activated aerolysin to selectively permeabilize the host plasma membrane. Following removal of excess toxin, temperature was shifted to 37 °C to induce membrane pore formation. Released schizonts were separated from host cell debris using Percoll density gradient centrifugation.

### 2.8 ICP-MS (inductively coupled plasma mass spectrometry)

To quantify intracellular trace metal concentrations, purified schizonts were subjected to ICP-MS analysis. Briefly, schizonts were washed 3 times with PBS, followed by the addition of 6 mL HNO_3_ and 3 mL H_2_O_2_. The mixture was allowed to stand for 1 h and digested using ETHOS UP microwave digestion system (Milestone, Italy). The vessels were subjected to three different digestion conditions: temperature (°C), hold times (min), and ramp times (°C/min) were adjusted at 120/15/5, 180/10/5 and 210/10/15, respectively (Cao et al., 2020). After cooling, the digested solution was collected and diluted to a final volume of 10 mL with 1% HNO_3_ for subsequent analysis using ICP-MS (Agilent, USA) to obtain total parasites Cu, Zn, Mn and Fe content.

### 2.9 ROS activity assays

TaNM cells (1 × 10^5^) were seeded in 24-well plates and cultured overnight incubated at 37 °C with 5% CO_2_. The medium was removed, and cells were washed twice with HBSS. Cells were incubated with dye working solution (DOJINDO, Japan) for 30 min at 37 °C in 5% CO_2_. After incubation, the dye solution was discarded, and cells were washed twice with HBSS. Cells were subsequently treated with PBT2 at final concentrations of 0.5 μM or 1 μM for 1 h or 2 h at 37 °C in 5% CO_2_. Following treatment, cells were washed twice with HBSS, resuspended in HBSS, and analyzed by flow cytometry (Beckman, USA).

### 2.10 Superoxide dismutase (SOD) activity assays

TaNM cells (5 × 10^6^) were treated with PBT2 at concentrations of 0.5 μM or 1 μM for 1 h or 2 h. Cells were harvested, washed once with ice-cold PBS, and centrifuged at 800×*g* for 2 min. The supernatant was discarded, and the cell pellet was resuspended in 1 mL of ice-cold 1 × lysis buffer. Cells were lysed by sonication (300 W) on ice for 3 min. The lysates were centrifuged at 12000×*g* for 5 min at 4 °C, and the supernatants were collected. SOD activity was measured using a commercial SOD assay kit according to the manufacturer's instructions (Abbkine). Briefly, appropriate volumes of samples, Working Xanthine Oxidase, Sample Diluent, and Working Reagent were added to a 96-well plate and mixed thoroughly. After incubation at 37 °C for 30 min, the absorbance was measured at 450 nm, and the SOD activity was calculated.

### 2.11 In vivo efficacy of PBT2 against growth of *B. microti* in infected mice

Four groups of mice (n = 5 per group) were injected intraperitoneally with 1 × 10^6^ *B. microti*-infected red blood cells (RBCs) except for the blank control group. When infected mice demonstrated 1% parasitemia (after 4 days), the mice were treated daily with each compound for 5 days. The second group mice were injected with PBS as the NC group, and the third group mice were treated with DA intraperitoneal at a dosage of 30 mg/kg as the PC group. The fourth and fifth groups mice were treated with PBT2 intraperitoneal at a dosage of 15 and 30 mg/kg. Every 2 days until the 24th day, blood collected was used to prepare Giemsa-stained thin smears. The parasitemia was calculated by observing the smears under a microscope and counting the infected RBCs among 2000 R BCs.

Approximately 20 μL of blood was collected from each mouse every 4 days via tail-tip bleeding. The RBCs number, hemoglobin (HGB) and hematocrit (HCT) were determined using the LinCyto automatic hematology analyzer (GlinX Biotechnology, China). After the study was completed, all the mice were euthanized humanely via inhalation of the chloroform, and dislocation of the neck.

### 2.12 Statistical analysis

All statistical tests were conducted using Prism version 9.0. Statistical significance was calculated by ANOVA in one way or two ways with multiple comparisons test.

## 3 Results

### 3.1 PBT2 effectively reduced *T. annulata* in vitro

RT-qPCR analysis demonstrated that treatment with 1 μM PBT2 for 24 h significantly reduced parasite gene expression in both TaNM and TaXJS cells (Fig. 1B). In TaNM cells, PBT2 decreased *TaSP* and *TaP104* transcript levels by approximately 83% and 71%, respectively, relative to DMSO control. Bup (600 nM) produced a comparable reduction in TaNM cells, suppressing *TaSP* and *TaP104* expressions by approximately 87% and 73%, respectively. In contrast, in TaXJS cells, Bup resulted in only minor reductions in *TaSP* (9%) and *TaP104* (13%) expression compared with DMSO control. However, PBT2 significantly decreased *TaSP* and *TaP104* expression by approximately 78% and 68%, respectively, in TaXJS cells.

**Fig. 2:**
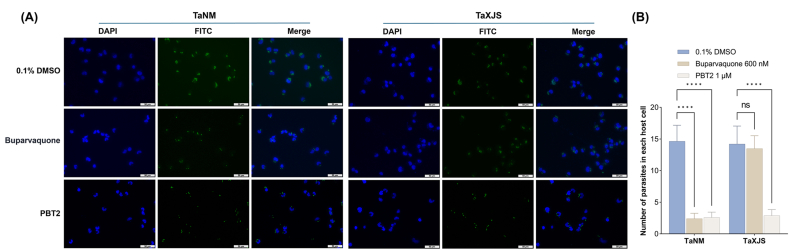
PBT2 reduces the schizont burden in *T. annulata*-infected cells. Representative immunofluorescence images of TaNM and TaXJS cells in each treatment group, schizont nuclei are shown in green and host cell nuclei in blue (A). Quantification of the number of schizonts per host cell in each treatment group (B). Error bars represent the SD of the mean from three independent biological replicates. ns, *P*> 0.05; ∗∗∗∗, *P* < 0.0001 by two-way ANOVA followed by Dunnett's multiple comparisons test.

We assessed the number of parasites in each host cell by counting 1000 cells per group following parasite specific nuclear staining (Fig. 2A). In TaNM cells, Bup and PBT2 reduced the parasite burden by approximately 83.5% and 82.3%, respectively, compared with the DMSO control. In TaXJS cells, Bup resulted in only a minimal reduction (5.1%) compared to the DMSO control, whereas PBT2 markedly decreased the number of schizonts per cell by approximately 79.7% (Fig. 2B).

### 3.2 PBT2 inhibits the proliferation of TaNM and TaXJS cells in vitro

**Fig. 3:**
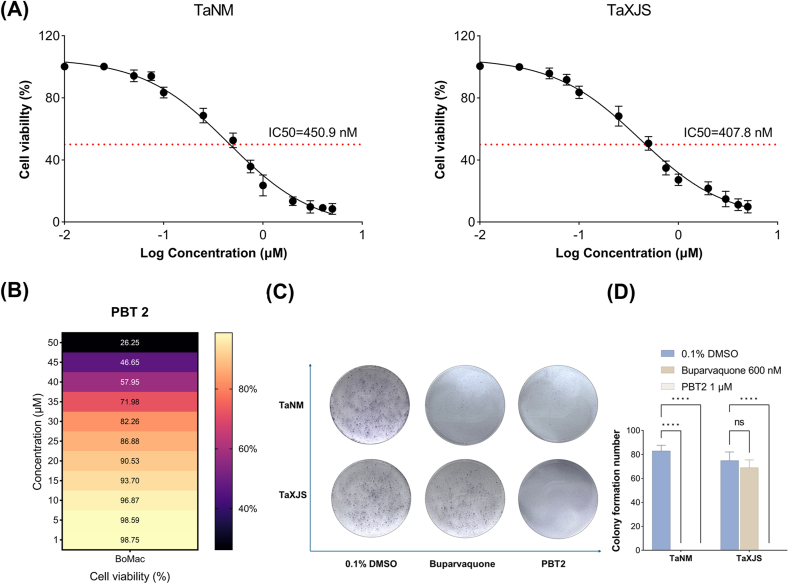
PBT2 inhibits the proliferation of TaNM and TaXJS cells in vitro. The IC_50_ values of PBT2 on TaNM and TaXJS cells (A). The cell viability of BoMac cells with different concentration of PBT2 (B). Colony formation assay demonstrated that PBT2 completely abolished clonogenic growth, representative images of colony formation are shown (C). Quantification of colony numbers before and after treatment with PBT2 or Bup (D). The values obtained were used to determine the IC_50_ using nonlinear regression (curve fitting analysis) in GraphPad Prism software. Error bars represent the SD of the mean from three independent biological replicates. ns, *P*> 0.05; ∗∗∗∗, *P* < 0.0001 by two-way ANOVA followed by Dunnett's multiple comparisons test.

Different concentrations of PBT2 were evaluated the effect on proliferation of TaNM and TaXJS cells for 48 h. The IC_50_ values of PBT2 on TaNM and TaXJS cells were 450.9 and 407.8 nM, respectively (Fig. 3A). The cell viability of BoMac cells was greater than 80 % when the concentration of PBT2 at 30 μM, indicating the cytotoxicity was low (Fig. 3B). The colony growth assay indicates that the TaNM cells were completely blocked by Bup (600 nM) and PBT2 (1 μM). Treatment with PBT2 (1 μM) was also able to inhibit the colony growth of TaXJS cells, in contrast, treatment with Bup (600 nM) had no effect on colony growth of TaXJS cells (Fig. 3C and D).

### 3.3 PBT2 disrupts metal ion homeostasis in *T. annulata*

**Fig. 4:**
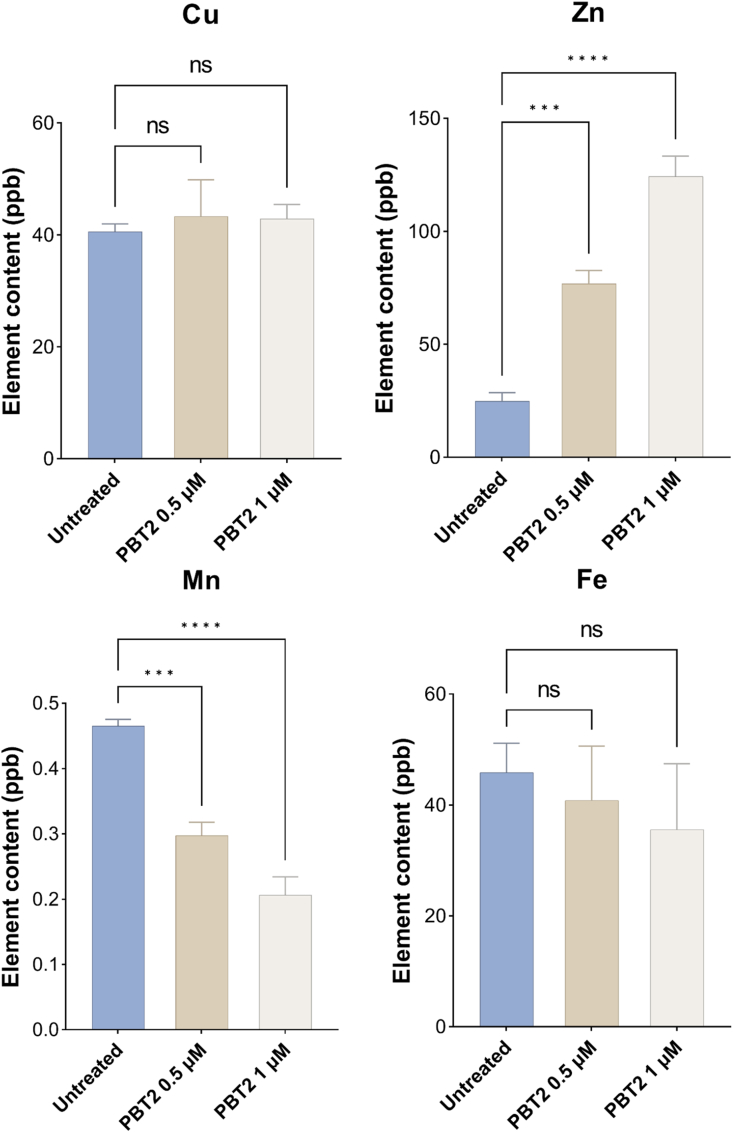
Metal ion content in *T. annulata* schizonts following PBT2 treatment. Error bars represent the SD of the mean from three independent biological replicates. ns, *P*> 0.05; ∗∗∗, *P*< 0.001; ∗∗∗∗, *P* < 0.0001 by one-way ANOVA followed by Dunnett's multiple comparisons test.

Metal ion concentrations in *T. annulata* schizonts following PBT2 treatment were quantified by ICP-MS. Two concentrations of PBT2 (0.5 and 1 μM) were evaluated (Fig. 4). Zn and Mn levels were significantly altered in response to PBT2 exposure. Specifically, intracellular Zn content increased in a concentration-dependent manner, whereas Mn content decreased as the concentration of PBT2 increased. In contrast, no significant changes were observed in Cu or Fe levels compared with the control group. These results indicate that PBT2 selectively perturbs Zn and Mn homeostasis in *T. annulata* schizonts.

### 3.4 PBT2 disrupts redox homeostasis and reduces SOD activity in infected cells

**Fig. 5:**
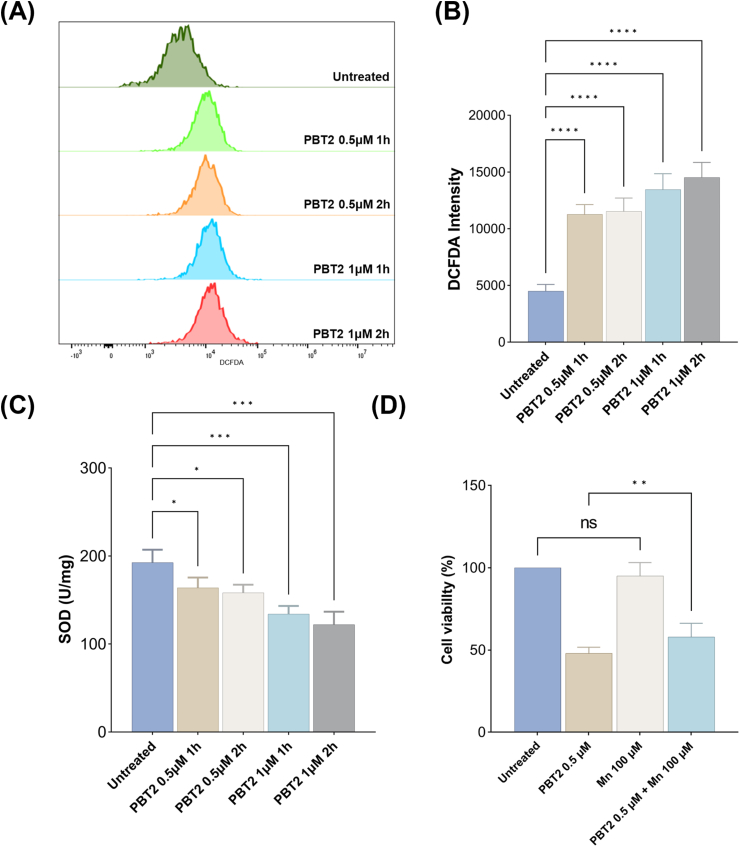
PBT2 disrupts redox homeostasis and reduces SOD activity in infected cells. ROS levels in TaNM cells following PBT2 treatment were quantified by flow cytometry, representative flow cytometry histograms are shown (A). Quantitative analysis of ROS levels (B). SOD activity in TaNM cells after PBT2 exposure, showing a significant reduction compared with untreated controls (C). Manganese supplementation partially rescued PBT2-induced growth inhibition in TaNM cells, indicating functional involvement of Mn depletion in redox imbalance (D). Error bars represent the SD of the mean from three independent biological replicates. ns, *P*> 0.05; ∗, *P*< 0.05; ∗∗, *P*< 0.005; ∗∗∗, *P*< 0.001; ∗∗∗∗, *P* < 0.0001 by one-way ANOVA followed by Tukey's multiple comparisons test.

We assessed ROS levels in TaNM cells, across two concentrations of PBT2 (0.5 μM and 1 μM) and two exposure durations (1 h and 2 h), ROS levels increased significantly in a dose and time dependent manner (Fig. 5A and B). This aligns with prior bacterial studies showing that intracellular zinc accumulation perturbes redox balance, leading to ROS generation (Bohlmann et al., 2018).

Concomitantly, the SOD activity in PBT2-treated cells exhibited a progressive decline (Fig. 5C). This dynamic suggests an initial compromise of antioxidant defenses, possibly through metal dependent enzyme inactivation and subsequent cellular adaptation. In bacterial systems, PBT2 induced zinc intoxication has been shown to inhibit manganese dependent SOD activity, both by depleting essential Mn^2+^ cofactors and by mismetallation, further exacerbating ROS accumulation (Harbison-Price et al., 2020).

To evaluate the functional relevance of Mn^2+^ depletion, we performed metal rescue experiments. Supplementation with exogenous Mn partially mitigated the decrease in cell viability induced by PBT2 (Fig. 5D), indicating that restoration of manganese bioavailability can ameliorate PBT2's effect. This rescue phenomenon is consistent with observations in *Streptococcus uberis* where Mn pre-treatment protected cells against PBT2-zinc lethality, presumably by reestablishing manganese dependent antioxidant capacity (Harbison-Price et al., 2020).

Collectively, these data indicate that PBT2 disrupts parasite metal homeostasis elevating Zn^2+^ and depleting Mn^2+^ thereby promoting oxidative stress and impairing Mn dependent antioxidant defenses, ultimately contributing to growth inhibition.

### 3.5 Effect of PBT2 on *B. microti* in mice

**Fig. 6:**
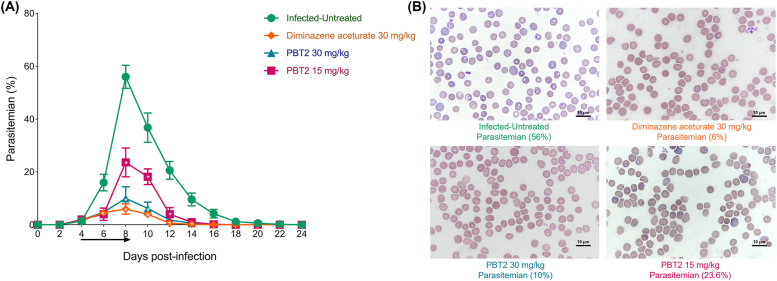
The growth inhibition of PBT2 on *B. microti* in vivo. Parasitemia was calculated by counting infected RBCs among 2000 R BCs using Giemsa-stained thin blood smears. The arrow indicates 5 consecutive days of treatment. Error bars represent the SD of the mean from five independent biological replicates, ∗, *P* < 0.05 by two-way ANOVA followed by Dunnett's multiple comparisons test (A). Representative Giemsa-stained blood smear image obtained at peak parasitemia on the 8th day (B).

**Fig. 7:**
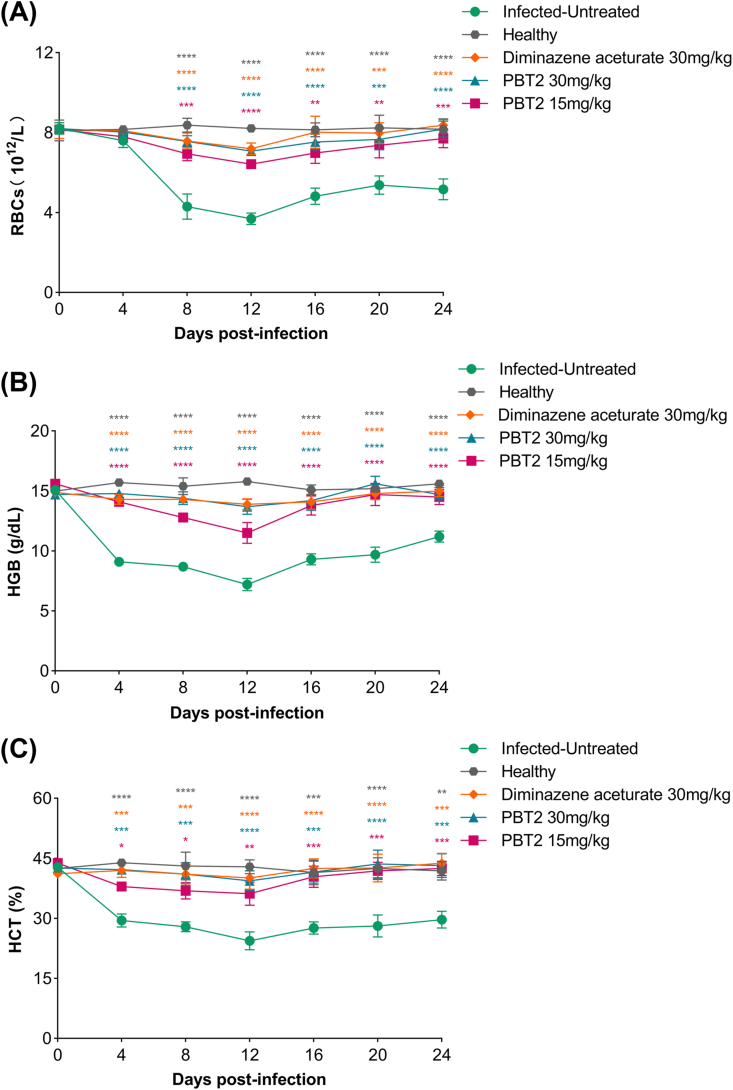
Hematology profiles of PBT2-treated mice in vivo. The changes of RBCs (A), HGB (B), and HCT (C) in mice treated with PBT2 and DA. Error bars represent the SD of the mean from five independent biological replicates. Different colored asterisks indicate that the differences between the various groups are statistically significant. ∗, *P*< 0.05; ∗∗, *P*< 0.005; ∗∗∗, *P*< 0.001; ∗∗∗∗, *P* < 0.0001 by two-way ANOVA followed by Dunnett's multiple comparisons test.

The inhibitory effect of PBT2 on *B. microti* was evaluated in a mice model. The parasitemia in DA-treated and PBT2-treated groups were less than the infected-untreated group at 6-12 days (Fig. 6A). The peak parasitemia level reached 56% (infected-untreated group), 6% (DA 30 mg/kg treated group), 10% (PBT2 30 mg/kg treated group) and 23.6% (PBT2 15 mg/kg treated group) on the 8th day (Fig. 6B). The number of RBCs (Fig. 7A), HGB concentration (Fig. 7B) and HCT percentage (Fig. 7C) in infected-untreated groups were all lower than the DA-treated and PBT2-treated groups in the 4-24 days.

## 4 Discussion

Piroplasm, including *Theileria* spp. and *Babesia* spp., continue to cause substantial economic losses in livestock production worldwide, and the increasing prevalence of drug resistance complicates disease control (Seangseerattanakulchai and Piratae, 2021). Consequently, the development of novel therapeutics is an urgent priority. PBT2, an orally bioavailable copper and Zn ionophore (Adlard et al., 2008), emerges as a promising candidate. In this study, we demonstrate PBT2 exerts inhibitory activity against *T. annulata* in vitro and *B. microti* in vivo, including activity against a Bup-resistant strain.

In vitro, PBT2 significantly reduced parasite specific transcripts (*TaSP* and *TaP104*) and decreased schizont burden in *T. annulata*-infected cells. Notably, PBT2 retained efficacy in TaXJS cells where Bup showed minimal activity. These findings suggest that PBT2 acts through a mechanism distinct from that of hydroxynaphthoquinones, which primarily target parasite mitochondrial electron transport. The similar IC_50_ values in TaNM and TaXJS cells suggest that resistance to Bup does not confer reduced susceptibility to PBT2. Importantly, PBT2 inhibited colony formation in both lines, whereas Bup failed to suppress clonogenic growth in the resistant strain. Together, these data indicate that PBT2 exerts a cytostatic effect linked to parasite burden reduction rather than nonspecific host toxicity.

Although PBT2 exhibited a favorable selectivity index (>60-fold) in BoMAC cells, its mechanism as a metal ionophore warrants careful consideration of potential host effects. Ionophores are capable of redistributing transition metals such as Zn^2+^ and Cu^2+^ across biological membranes, thereby altering intracellular metal availability (Adlard et al., 2008). Sustained perturbation of metal homeostasis has been shown to interfere with metalloenzyme function and mitochondrial redox regulation in mammalian systems (Sensi et al., 2009; Maret, 2013). Therefore, although acute cytotoxicity may be limited, prolonged dysregulation of transition metal homeostasis could theoretically affect host metabolic and antioxidant pathways. In our study, BoMAC viability remained above 80% at 30 μM well above the anti-parasitic IC_50_ values indicating limited cytotoxicity under the tested conditions. These observations are consistent with previous preclinical and clinical evaluations reporting acceptable tolerability of PBT2 in mammalian systems (Lannfelt et al., 2008; Caballero et al., 2016).

Trace metal balance has been implicated in the pathophysiology of bovine theileriosis. Recent clinical work demonstrated that adjunctive injectable trace mineral supplementation improved hematological and biochemical recovery in calves naturally infected with *T. annulata* (Ram et al., 2026), suggesting that modulation of metal availability can influence host-parasite interactions in vivo. Nevertheless, extrapolation of PBT2 use to bovine hosts requires systematic evaluation of pharmacokinetics, trace metal distribution, and hematological parameters during prolonged exposure to define a safe therapeutic window under field conditions.

While the precise anti-parasitic mechanism of PBT2 remains under investigation, its function as a Cu/Zn ionophore provides a strong mechanistic hypothesis (Harbison-Price et al., 2020). ICP-MS analysis revealed that PBT2 treatment altered metal ion homeostasis in *T. annulata* schizonts. Specifically, Zn content within schizonts increased in a dose-dependent manner, whereas Mn content decreased. In contrast, Cu and Fe levels did not change significantly under the tested conditions. These data indicate that PBT2 selectively perturbs Zn and Mn homeostasis at the parasite level rather than inducing a broad, nonspecific disruption of transition metals.

Zn-driven Mn depletion has been described in several bacterial pathogens, where elevated intracellular Zn competitively interferes with Mn acquisition systems, resulting in functional Mn limitation (McDevitt et al., 2011; Eijkelkamp et al., 2014). Although the metal transport machinery of *Theileria* is not yet well characterized, the reciprocal change in Zn and Mn observed in purified schizonts is compatible with a model in which Zn accumulation functionally reduces Mn availability within the parasite. Further studies will be required to determine whether this reflects competitive transport, altered metal binding equilibria, or secondary redistribution mechanisms.

Given the established role of Mn as an essential cofactor for SOD (Chen et al., 2018), we next assessed redox parameters following PBT2 exposure. Because direct ROS measurement in purified schizonts was technically limited, ROS levels and total SOD activity were quantified in infected cells. PBT2 treatment induced a dose and time dependent increase in ROS, accompanied by a sustained reduction in total SOD activity.

Although the assay measured overall SOD activity rather than Mn specific SOD isoforms, the concurrent depletion of Mn in purified schizonts and the partial restoration of cell viability upon Mn supplementation support a functional link between Mn availability and redox imbalance. In microbial systems, PBT2-mediated Zn accumulation has been shown to disrupt Mn-dependent SOD function through Mn depletion and mismetallation (Bohlmann et al., 2018).

The evaluation of novel compounds for *T. annulata* is challenging due to the practical difficulties of conducting drug trials in its natural bovine host. To circumvent this limitation, we utilized the well-established *B. microti*-mouse model to investigate the in vivo efficacy of PBT2 (Batiha et al., 2019). Both *Babesia* and *Theileria* are members of the apicomplexan phylum (Piroplasmida) and share conserved features of intraerythrocytic development and core metabolic processes, including reliance on redox balance and mitochondrial function (Hikosaka et al., 2010; Sojka et al., 2022). Consequently, demonstration of in vivo activity in the *B. microti* model provides supportive evidence that PBT2 exerts systemic anti-piroplasm effects in a mammalian host context.

In the present study, PBT2 treatment significantly delayed the rise in parasitemia between days 6 and 12 post-infection compared with untreated controls. Peak parasitemia on day 8 reached 56% in infected-untreated mice, compared with 6% in the DA (30 mg/kg) group and 10% and 23.6% in mice receiving 30 mg/kg and 15 mg/kg PBT2, respectively. The greater suppression observed at 30 mg/kg PBT2 indicates a dose-dependent effect. Although complete parasite clearance was not achieved at the tested doses, the magnitude of parasitemia reduction suggests biologically relevant in vivo activity. As dose–response relationships are well documented in murine *B. microti* models (Mordue and Wormser, 2019), incomplete parasite clearance may be due to insufficient drug levels achieved with the current dosing regimen.

Beyond parasitemia control, PBT2 treatment attenuated infection-associated anemia. Infected-untreated mice exhibited marked reductions in RBC count, HGB, and HCT, whereas these parameters were partially restored in treated groups. This improvement likely reflects reduced parasite burden and suggests that PBT2 confers measurable physiological benefit during infection.

Nevertheless, important biological differences must be acknowledged. *B. microti* is confined to erythrocytes, whereas *T. annulata* develops a schizont stage in bovine leukocytes and induces host-cell transformation (Dobbelaere and Heussler, 1999; Vannier et al., 2008). Consequently, the murine *B. microti* model does not reproduce the leukocyte transformation characteristic of *T. annulata* infection in cattle. These findings support systemic anti-piroplasm activity in vivo but do not establish efficacy in the natural bovine host. Further pharmacokinetic and efficacy studies in cattle are required.

In conclusion, PBT2 represents a novel paradigm for anti-hemoparasite drug discovery: exploiting metal ion biology to selectively disrupt essential parasite metabolism. Our findings demonstrate its promising efficacy, favorable safety window in relevant models, and activity against drug-resistant strains, warranting further development for the control of economically devastating piroplasmid infections.

## 5 Conclusion

Collectively, our findings demonstrate that PBT2 represents a novel and effective anti-*T. annulata* agent in vitro and confers potent anti-*B. microti* activity in vivo. The compound's distinct metal ionophore pharmacology suggests broad potential as an anti-piroplasm therapy, warranting further investigation against related hemoparasites.

## CRediT authorship contribution statement

**Jin Che:** Writing – original draft, Formal analysis. **Yixuan Wu:** Writing – original draft. **Junwei Wang:** Methodology. **Yijun Chai:** Resources. **Jinming Wang:** Conceptualization. **Wei Li:** Resources. **Shuaiyang Zhao:** Writing – review & editing. **Guiquan Guan:** Supervision, Funding acquisition. **Hong Yin:** Supervision, Funding acquisition.

## Consent for publication

Not applicable.

## Data statement

All relevant data are within the manuscript.

## Ethics approval

In the present study, all the animal experiments were approved by the Animal Ethics Committee of the Lanzhou Veterinary Research Institute, Chinese Academy of Agricultural Sciences. All experimental animals used were dealt with according to the Animal Ethics Procedures and Guidelines of the People's Republic of China (LVRIAEC-2024-034).

## Funding statement

The study was financially supported by the 10.13039/501100012166National Key Research and Development Program of China (2024YFD1800100), the Innovation Program of 10.13039/501100005196Chinese Academy of Agricultural Sciences (CAAS-ASTIP-2021-LVRI), NBCITS (CARS-37), National Parasitic Resources Center (NPRC-2019-194-30), the Science Fund for Creative Research Groups (22JR5RA024) and Special Project (22CX8NA011) of Gansu Province.

## Competing interest

The authors declare that there is no conflict of interest.
